# Genetically predicted circulating polyunsaturated fatty acids and Crohn’s disease susceptibility: A two-sample mendelian randomization study

**DOI:** 10.1097/MD.0000000000050897

**Published:** 2026-09-25

**Authors:** Tingting Zhu, Qiang Hu, Kaifeng Zhu

**Affiliations:** a Department of Nursing, Tongde Hospital of Zhejiang Province, Hangzhou, China; b The Integrated Traditional Chinese and Western Medicine School of Clinical Medicine, Zhejiang Chinese Medicine University, Hangzhou, China; c Department of general surgery, Tongde Hospital of Zhejiang Province, Hangzhou, China.

**Keywords:** Crohn disease, Mendelian randomization, omega-3 fatty acids, omega-6 fatty acids, omega-6/omega-3 ratio

## Abstract

Crohn disease (CD) remains an increasing global health burden. Omega-3 and omega-6 polyunsaturated fatty acids participate in lipid metabolism and inflammatory regulation, but previous observational and interventional studies have produced inconsistent findings. This study aimed to assess whether genetically predicted circulating omega-3, omega-6, and the omega-6/omega-3 ratio are associated with CD susceptibility using a two-sample Mendelian randomization (MR) design. Genome-wide association study summary data for circulating omega-3, omega-6, and the omega-6/omega-3 ratio were obtained from the IEU Open GWAS project (each n = 114,999), while CD outcome data included 12,194 cases and 28,072 controls. Genome-wide significant single-nucleotide polymorphisms (*P* < 5 × 10^−8^) were clumped at *r*^2^ = 0.001 within 10,000 kb, and all retained instruments had F-statistics > 10. Multiplicative random-effects inverse-variance weighting was used as the primary analysis, supplemented by MR-Egger, weighted median, simple mode, and weighted mode methods. Heterogeneity, horizontal pleiotropy, and robustness were evaluated using Cochran’s *Q* test, the MR-Egger intercept, leave-one-out analysis, and funnel plots. A total of 46 single-nucleotide polymorphisms for omega-3, 51 for omega-6, and 34 for the omega-6/omega-3 ratio were included. Higher genetically predicted circulating omega-3 levels were associated with lower odds of CD (odds ratio [OR] = 0.823, 95% confidence interval [CI] 0.721–0.939, *P* =.004), whereas omega-6 showed no statistically significant association (OR = 0.885, 95% CI 0.727–1.061, *P* =.186). A higher genetically predicted omega-6/omega-3 ratio was associated with increased odds of CD (OR = 1.211, 95% CI 1.069–1.372, *P* =.003). Complementary MR methods showed generally concordant directions, and MR-Egger intercept tests provided no statistical evidence of directional horizontal pleiotropy. However, substantial between-instrument heterogeneity was observed for all three exposures. These findings suggest that genetically predicted higher circulating omega-3 levels and a lower omega-6/omega-3 ratio are associated with lower CD susceptibility, whereas omega-6 alone shows no statistically significant association. Given the substantial between-instrument heterogeneity, these results should be regarded as suggestive genetic evidence rather than definitive proof of causality and should not be interpreted as evidence that dietary omega-3 intake or supplementation prevents CD.

## 1. Introduction

Crohn’s disease (CD) is a chronic, relapsing inflammatory bowel disorder that can affect any segment of the digestive tract and is characterized by cramp-like abdominal pain, persistent diarrhea, weight loss, and a high rate of complications. The Global Burden of Disease 2021 study estimated that about 5.8 million people worldwide were living with CD in 2021. Although incidence has leveled off in North America and Europe over the past two decades, it continues to rise rapidly in Asia and Latin America.^[1]^ The disease imposes a substantial burden on quality of life, work capacity, and health-care costs, making the early identification of modifiable risk factors and the implementation of primary-prevention strategies increasingly important.

Diet is widely regarded as a key external trigger that can initiate or exacerbate CD. Within the polyunsaturated fatty acid (PUFA) family, omega-3 and omega-6 lipids modulate eicosanoid production, NF-κB signaling, and intestinal barrier function, thereby shaping the balance between mucosal inflammation and epithelial repair.^[2,3]^ Long-chain omega-3 PUFAs, including docosahexaenoic acid (DHA) and eicosapentaenoic acid (EPA), are generally considered anti-inflammatory, immunomodulatory, and protective of the epithelium, whereas certain omega-6 PUFAs, particularly arachidonic acid, can be metabolized into pro-inflammatory mediators.^[4,5]^ Recent in vitro and animal studies, however, suggest that the upstream omega-6 precursor linoleic acid can, at specific concentrations, suppress pro-inflammatory cytokine release, indicating that its effects are not uniformly pro-inflammatory.^[6]^

Epidemiological findings are similarly inconsistent. Large prospective cohorts have reported an inverse association between marine-derived omega-3 intake and CD risk, yet randomized trials of fish oil supplements have failed to replicate this protective signal.^[7]^ High dietary omega-6 intake or an imbalanced omega-6/omega-3 ratio has been implicated in CD progression by some studies, whereas others have found no significant association.^[8]^ Notably, the omega-6/omega-3 ratio may better capture the net effect of the fatty acid spectrum on mucosal immune and inflammatory networks than absolute intakes alone, but robust causal evidence for this ratio remains limited.

Traditional observational studies are vulnerable to confounding by diet, lifestyle, socioeconomic status, comorbidities, and disease-related changes in food intake. Mendelian randomization (MR) uses germline genetic variants associated with an exposure as instrumental variables and can reduce, but not eliminate, these sources of bias.^[9]^ The validity of causal interpretation depends on three assumptions: the genetic instruments must be associated with the exposure, must be independent of confounders of the exposure-outcome relationship, and must influence the outcome only through the exposure. Consequently, MR estimates should be interpreted as effects of genetically proxied exposure differences rather than as direct estimates of the effect of a specific dietary intervention.

Against this background, we integrated large GWAS datasets and applied a two-sample MR design to evaluate: whether genetically predicted circulating omega-3 levels are associated with CD susceptibility; whether genetically predicted circulating omega-6 levels are associated with CD susceptibility; and whether the genetically predicted omega-6/omega-3 ratio is associated with CD susceptibility. We aimed to provide genetic evidence relevant to PUFA biology in CD while explicitly distinguishing genetically influenced circulating PUFA levels from modifiable dietary intake or supplementation.

## 2. Materials and methods

### 2.1. Study design

We adopted a two-sample Mendelian-randomization (MR) framework to evaluate the association of genetically predicted circulating omega-3 fatty acids, omega-6 fatty acids, and the omega-6/omega-3 ratio with Crohn’s disease (CD) (Fig. 1). Three assumptions underpin MR (Fig. 2).^[10]^ First, the relevance assumption requires that the instrumental SNPs be robustly associated with the exposure. Second, the independence assumption requires that the instruments are not associated with confounders of the exposure–outcome relationship. Third, the exclusion-restriction assumption requires that the instruments influence CD only through the PUFA exposure being modeled, rather than through an alternative biological pathway. Because the latter two assumptions cannot be proven from GWAS summary data alone, we performed multiple sensitivity analyses and interpreted the findings cautiously.

**Figure 1.**
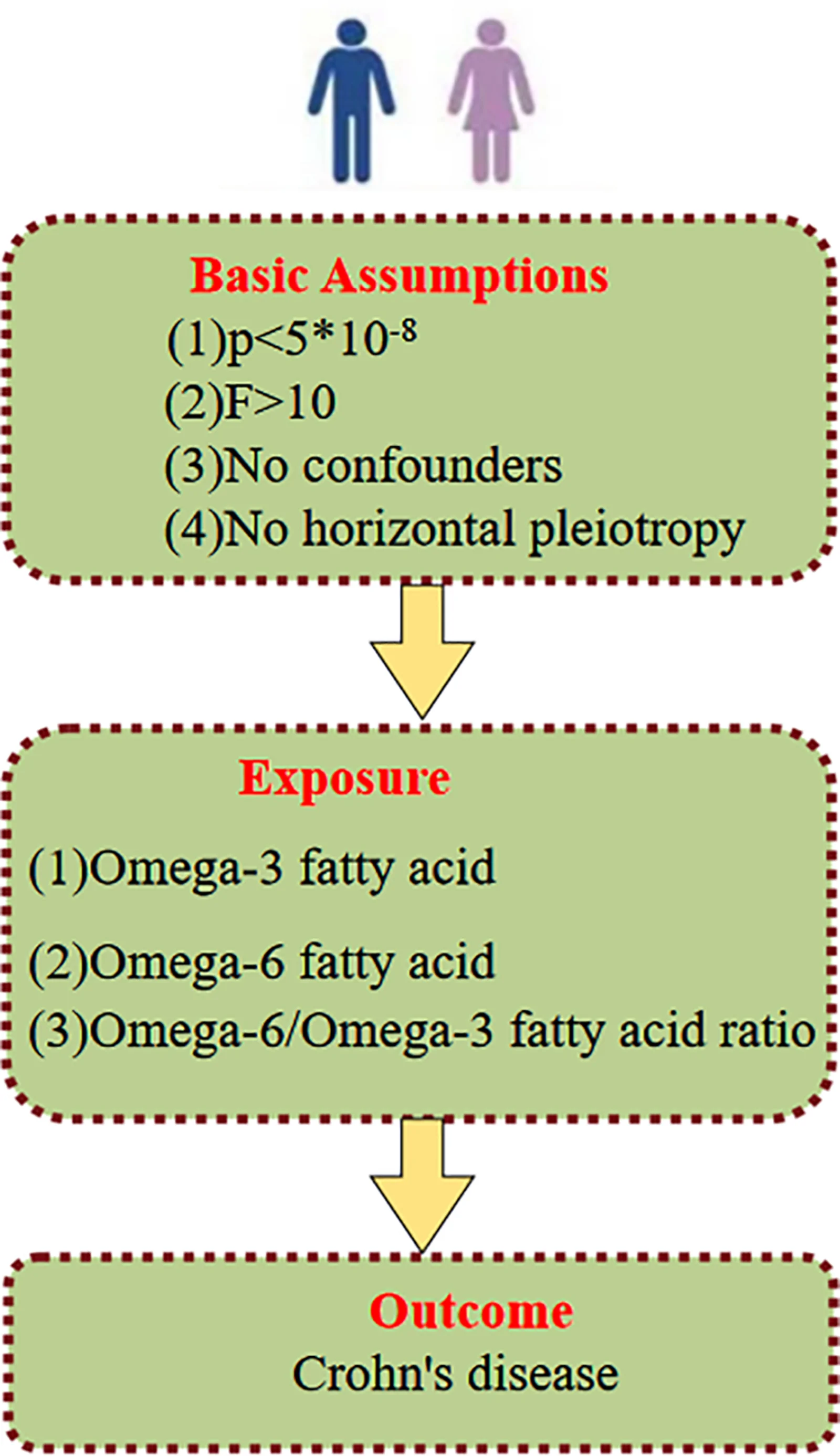
Flowchart of Mendelian randomization.

**Figure 2.**
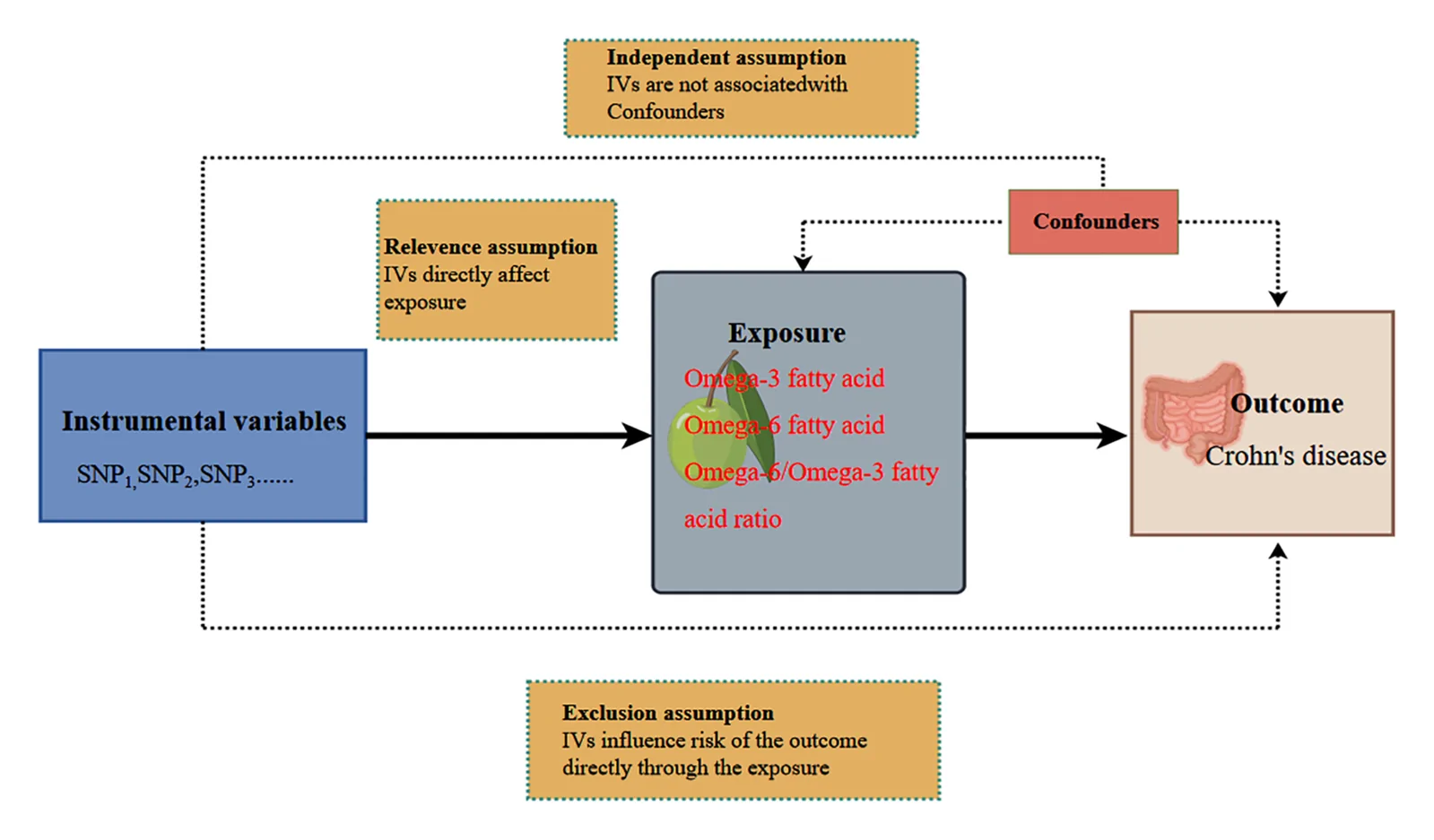
Flowchart of 3 assumptions.

### 2.2. Data sources

All summary-level statistics were obtained from the publicly accessible IEU Open GWAS Project. Circulating omega-3 levels were represented by the dataset “met-d-Omega_3” (n = 114,999; 12,321,875 SNPs), circulating omega-6 levels by “met-d-Omega_6” (n = 114,999; 12,321,875 SNPs), and the omega-6/omega-3 ratio by “met-d-Omega_6_by_Omega_3” (n = 114,999; 12,321,875 SNPs). These metabolomic GWAS data derive from large-scale UK Biobank analyses in which multiple genome-wide significant loci were identified for circulating PUFA traits.^[11]^ The CD outcome dataset was “ebi-a-GCST004132,” based on the International IBD Genetics Consortium and comprising 12,194 cases and 28,072 controls (40,266 participants; 9,457,998 SNPs).^[12]^ All participants were of European ancestry. Exposure and outcome summary data were obtained from separate GWAS resources to minimize sample overlap. Because the data were de-identified and publicly available, no additional ethics approval was required.

### 2.3. SNP selection

Instrumental variants were selected in R using the TwoSampleMR package. SNPs associated with each exposure at genome-wide significance (*P* < 5 × 10^−8^) were clumped using an LD threshold of r^2^ = 0.001 within a 10,000-kb window. Exposure and outcome alleles were then harmonized, and palindromic or incompatible variants that could not be aligned reliably were excluded. Instrument strength was assessed using F = β^2^/SE^2^; all retained variants had F > 10. The statistical association of these variants with circulating PUFA levels satisfies the MR relevance criterion but does not imply that every SNP acts directly on PUFA concentrations. Some variants may influence PUFA levels through fatty-acid desaturation, elongation, transport, or broader lipid-metabolic pathways; therefore, potential horizontal pleiotropy was evaluated in sensitivity analyses and considered in the interpretation.

### 2.4. Statistical methods

Data import, instrumental-variable selection, Mendelian-randomization (MR) analyses, and sensitivity tests were carried out with the “TwoSampleMR” package (version 0.6.15) in R 4.4.1.

#### 2.4.1. MR analysis

The primary analysis used the inverse-variance-weighted (IVW) estimator. In the TwoSampleMR package, the primary IVW analysis was implemented using a multiplicative random-effects model. Briefly, SNP-specific associations with CD were regressed on the corresponding SNP-exposure associations with the intercept constrained to zero, and each variant was weighted according to the inverse variance of its SNP-outcome association.^[10,13]^ This approach combines the individual Wald-ratio estimates across genetic instruments. The multiplicative random-effects IVW model allows for over-dispersion among SNP-specific estimates and adjusts the standard error of the pooled estimate when between-instrument heterogeneity is present. Importantly, this approach accounts for statistical heterogeneity in the uncertainty of the pooled estimate but does not identify the source of heterogeneity or eliminate bias arising from invalid instruments or horizontal pleiotropy. Therefore, MR-Egger regression, weighted median, simple mode, and weighted mode methods were used as complementary estimators. Effect estimates are reported as odds ratios (ORs) with 95% confidence intervals (CIs).

#### 2.4.2. Sensitivity analysis

Sensitivity analyses were performed to evaluate the robustness of the MR estimates and potential violations of the instrumental-variable assumptions. First, directional horizontal pleiotropy was assessed using the MR-Egger intercept test; a statistically significant nonzero intercept was considered evidence of average directional pleiotropy. Second, Cochran’s Q statistics were calculated under both IVW and MR-Egger models to assess between-instrument heterogeneity. A statistically significant Q statistic was interpreted as evidence that the SNP-specific estimates varied more than expected from sampling error alone. Such heterogeneity was considered an important limitation that reduces the certainty of interpreting the pooled estimate as a homogeneous causal effect. Third, leave-one-out analyses were performed by sequentially removing each SNP to determine whether the overall estimate was disproportionately driven by a single genetic variant. Fourth, funnel plots were inspected for marked asymmetry. Because these sensitivity analyses have limited ability to detect all forms of horizontal pleiotropy, particularly balanced or pathway-specific pleiotropy, concordance across methods was considered supportive but not definitive evidence for causality.

## 3. Results

### 3.1. Relationship between omega-3 fatty acids and Crohn’s disease

#### 3.1.1. Instrumental-variable results

A total of 52 SNPs that were strongly associated with the exposure and mutually independent were initially included. Five SNPs were lost during harmonization with the outcome dataset, and one palindromic or incompatible SNP (rs5112) was removed, leaving 46 SNPs for analysis (Table 1). All of these SNPs had *F* values >10; the *F*-value range was 26.163 to 6315.263, with a mean of 251.242, indicating no weak-instrument bias.

**Table 1:** A total of 46 independent SNPs strongly associated with the exposure were included for Omega-3 fatty acids.

SNP	effect	other	beta	se	chr	chr.1	eaf	pval	F_value
rs10184054	G	C	−0.0361462	0.00486548	2	2	0.224107	5.6E–15	55.19171777
rs10455872	G	A	−0.0629576	0.00753435	6	6	0.078988	2.8E–17	69.82400343
rs112875651	A	G	−0.0873719	0.00422001	8	8	0.392346	3.5E–98	428.663593
rs1132899	C	T	0.0270834	0.00410188	19	19	0.509603	8.6E–11	43.59537984
rs11563251	T	C	0.0349727	0.00647476	2	2	0.110601	0.000000032	29.1750074
rs1167998	A	C	0.0713574	0.00425038	1	1	0.644674	3.6E–66	281.8529057
rs11681659	T	C	−0.0251255	0.004486	2	2	0.716465	0.00000002	31.36973772
rs117143374	C	T	−0.0370966	0.005847	21	21	0.142254	2.2E–10	40.25334975
rs117733303	G	A	−0.115945	0.0150731	6	6	0.018513	1.4E–15	59.16963483
rs12226389	C	T	−0.050608	0.00524839	11	11	0.18582	1.1E–22	92.97927393
rs13424225	T	G	0.0221268	0.00408591	2	2	0.449809	0.000000022	29.32645904
rs139974673	C	T	0.117987	0.0128075	15	15	0.025918	2.3E–21	84.86714419
rs143355652	T	C	−0.154138	0.0204073	11	11	0.010467	9.4E–14	57.04904008
rs144018203	C	G	0.107134	0.02049	11	11	0.010653	0.000000042	27.33825082
rs1672811	C	T	0.0251849	0.0046967	16	16	0.748488	0.00000003	28.7537717
rs16940904	T	C	−0.0355285	0.0048772	17	17	0.226571	3.9E–14	53.06554317
rs174564	G	A	−0.337094	0.00424185	11	11	0.347013	1E–200	6315.262848
rs1800978	G	C	−0.0373414	0.00619432	9	9	0.123991	5.2E–09	36.34075269
rs182611493	G	A	−0.209571	0.0195777	19	19	0.012519	1.1E–27	114.5879719
rs261290	C	T	−0.114383	0.00428189	15	15	0.654653	3.9E–161	713.5952545
rs3018731	G	A	−0.0353357	0.00456603	11	11	0.717549	2E–14	59.88938826
rs34663616	A	C	0.0356728	0.00602277	15	15	0.137654	4.4E–10	35.08179779
rs35135293	T	C	−0.0208868	0.00408348	2	2	0.51675	0.000000039	26.16272346
rs4000713	A	G	−0.0288196	0.00446039	7	7	0.295408	1E–11	41.74747666
rs4860987	T	A	0.0463493	0.00491057	4	4	0.258609	1.2E–21	89.08868457
rs5112	G	C	0.0476852	0.00437178	19	19	0.533736	9.1E–30	118.9736044
rs55891451	C	A	0.0341853	0.00508075	10	10	0.201728	4.6E–12	45.27131809
rs58542926	T	C	−0.171666	0.00775231	19	19	0.074383	1.4E–113	490.3501511
rs6129624	A	G	−0.0257607	0.00437946	20	20	0.335237	5.1E–10	34.59984808
rs62466318	T	C	−0.0721329	0.00506371	7	7	0.204178	1.2E–45	202.9220001
rs629301	T	G	0.0382887	0.00488385	1	1	0.778033	1.3E–14	61.46340398
rs633695	G	A	0.0840069	0.00448287	15	15	0.292348	9.1E–80	351.1701782
rs6601924	C	T	0.0350603	0.00563873	10	10	0.845765	8.5E–10	38.66060592
rs6693447	G	T	0.0229488	0.00407772	1	1	0.461686	4.8E–09	31.67270528
rs673335	C	T	−0.0669996	0.00554146	11	11	0.159762	1.1E–34	146.1827119
rs6882345	A	G	0.0288844	0.00421159	5	5	0.632863	1.9E–13	47.03644923
rs72789541	A	T	−0.0810999	0.00446068	16	16	0.29597	5.6E–75	330.5510184
rs73109460	A	G	−0.0349475	0.006216	7	7	0.123622	9.2E–10	31.60896063
rs737338	T	C	−0.0726631	0.0110354	19	19	0.035186	3.5E–11	43.3562473
rs77960347	G	A	0.161749	0.0177574	18	18	0.013239	7.2E–22	82.97064255
rs7819706	G	A	−0.0396447	0.00628878	8	8	0.118291	1.8E–10	39.74087888
rs7924036	T	G	0.0233527	0.00406452	10	10	0.504205	5.5E–10	33.01077121
rs7970695	A	G	−0.0253039	0.00419603	12	12	0.620549	1.2E–10	36.36618603
rs9304381	T	C	0.0528854	0.00527814	18	18	0.818434	5.2E–24	100.3944665
rs964184	C	G	−0.116637	0.00596503	11	11	0.867229	8.9E–87	382.3379586
rs9987289	G	A	0.0566995	0.00707191	8	8	0.909151	3.2E–16	64.28135284

#### 3.1.2. MR analysis results

The multiplicative random-effects IVW analysis showed an inverse association between genetically predicted circulating omega-3 and CD [OR = 0.823, 95% CI 0.721–0.939, *P* = .004]. MR-Egger, weighted median, and simple mode estimates were directionally consistent and statistically significant (*P* < .05). These findings support an inverse association between genetically predicted circulating omega-3 and CD susceptibility; however, the strength of causal interpretation is limited by the substantial heterogeneity described below (Figs. 3 and 4).

**Figure 3.**
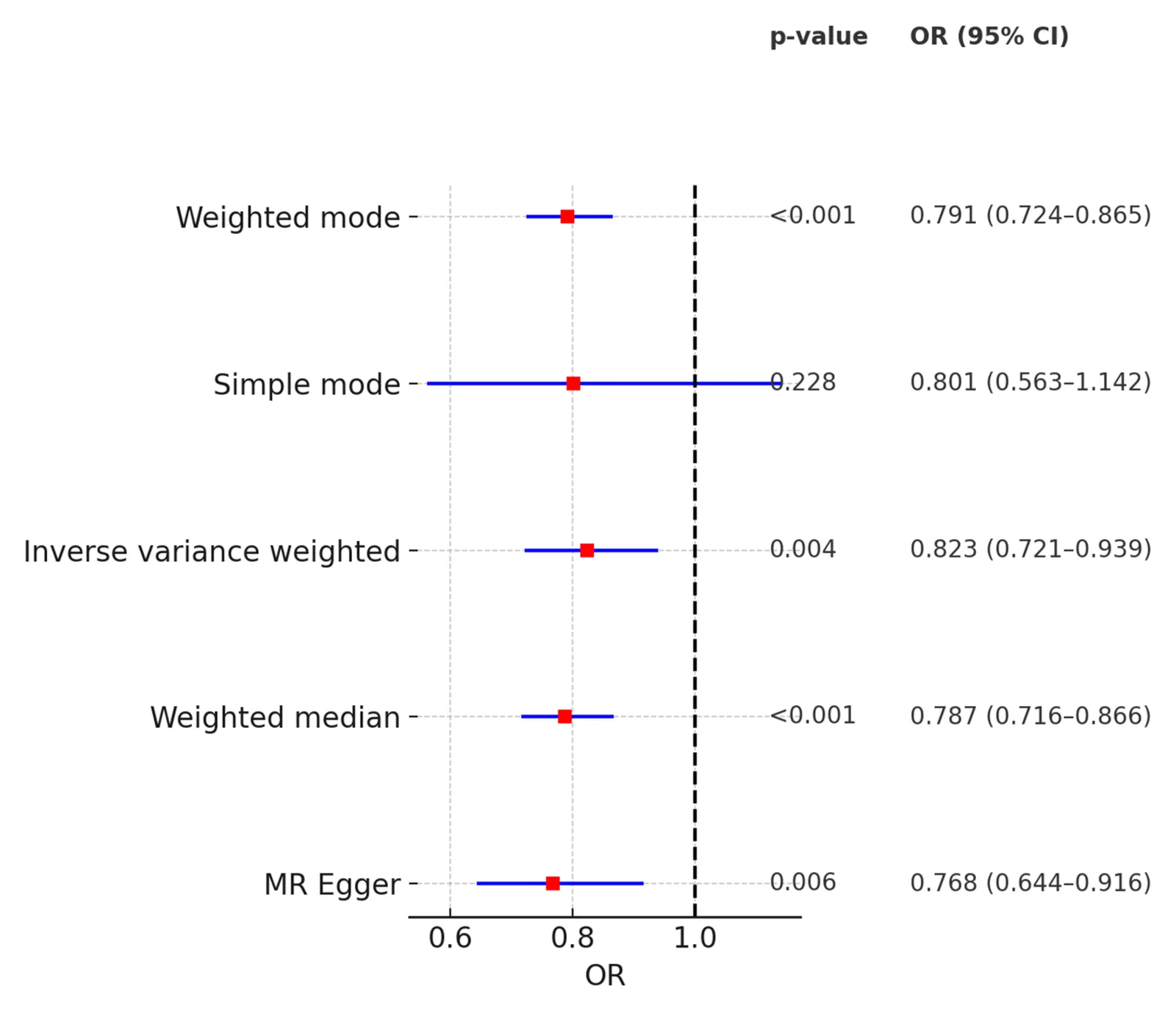
MR estimates for the association between genetically predicted omega-3 fatty acids and Crohn’s disease.

**Figure 4.**
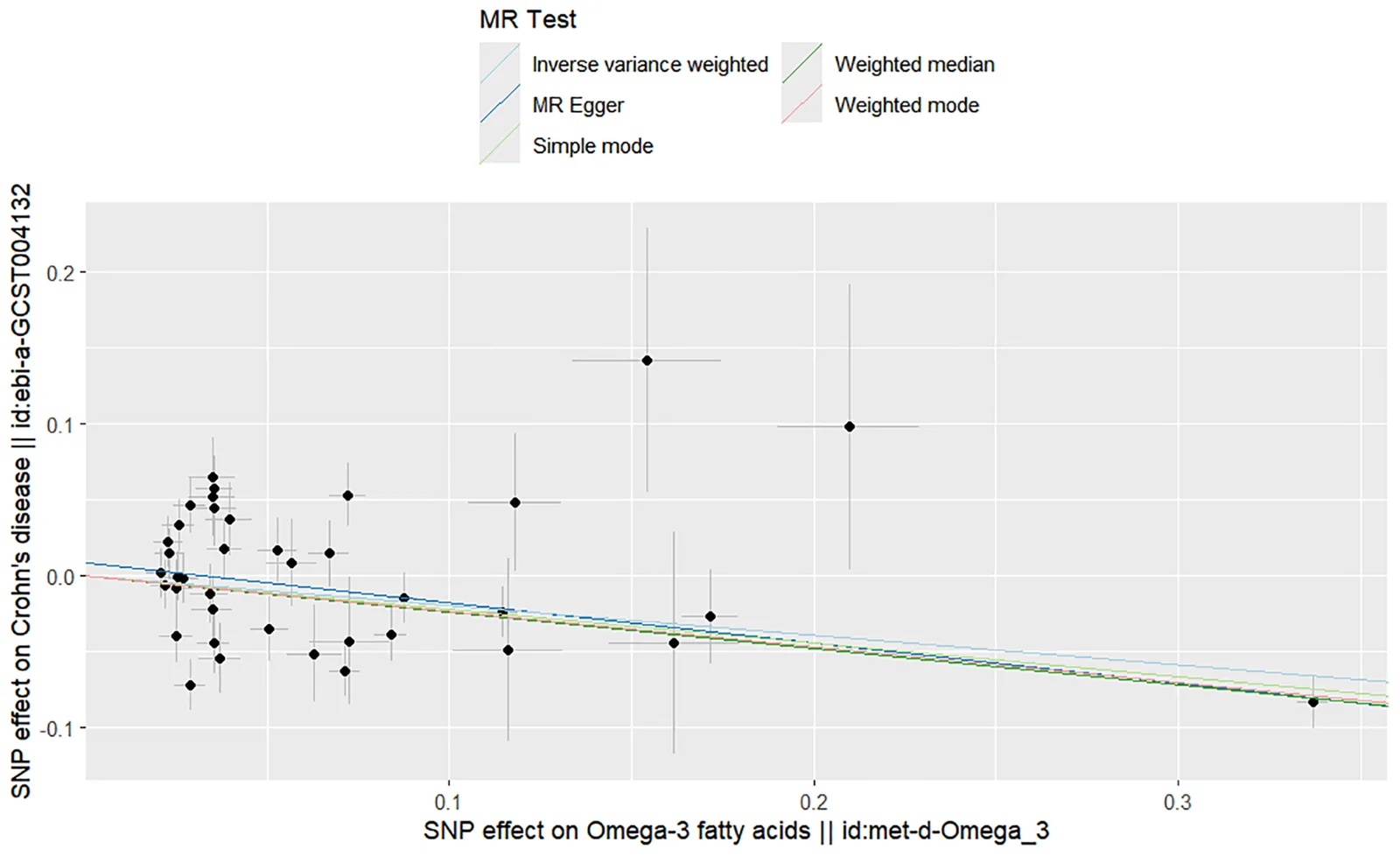
Scatter plot of the association between genetically predicted omega-3 fatty acids and Crohn’s disease.

#### 3.1.3. Sensitivity analysis

Cochran’s Q test showed substantial heterogeneity among the omega-3 instruments under both the IVW model (Q = 114.548, *P* < .001) and MR-Egger regression (Q = 110.620, *P* < .001). This significant between-instrument heterogeneity indicates that the SNP-specific estimates are not fully homogeneous and therefore reduces the certainty with which the pooled estimate can be interpreted as a single causal effect. We used a multiplicative random-effects IVW model, which allows for over-dispersion among SNP-specific estimates and adjusts the standard error of the pooled estimate in the presence of heterogeneity. However, this approach does not identify the source of heterogeneity and cannot eliminate bias caused by invalid instruments or horizontal pleiotropy. The MR-Egger intercept was 0.009 (*P* = .259), providing no statistical evidence of directional horizontal pleiotropy, although this test has limited power. Leave-one-out analysis showed that no single SNP disproportionately influenced the pooled association (Fig. 5), and the funnel plot was broadly symmetrical (Fig. 6). These sensitivity analyses provide some reassurance that the association was not driven by one obvious outlier or strong directional pleiotropy; nevertheless, balanced pleiotropy and heterogeneous biological pathways cannot be excluded. Therefore, the observed inverse association should be regarded as suggestive rather than definitive evidence of causality. The forest plot is shown in Figure 7.

**Figure 5.**
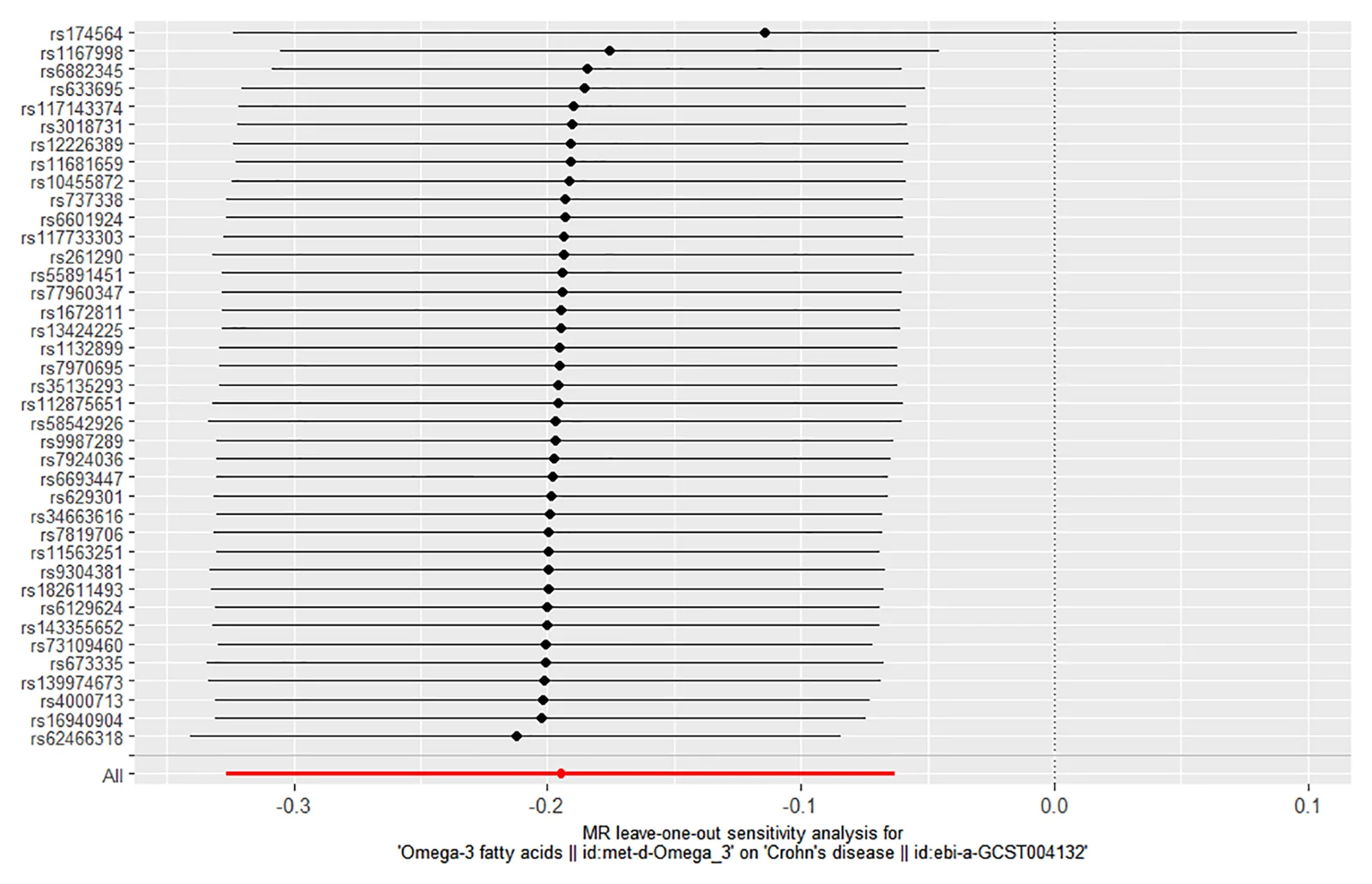
Leave-one-out analysis of the association between genetically predicted omega-3 fatty acids and Crohn’s disease.

**Figure 6.**
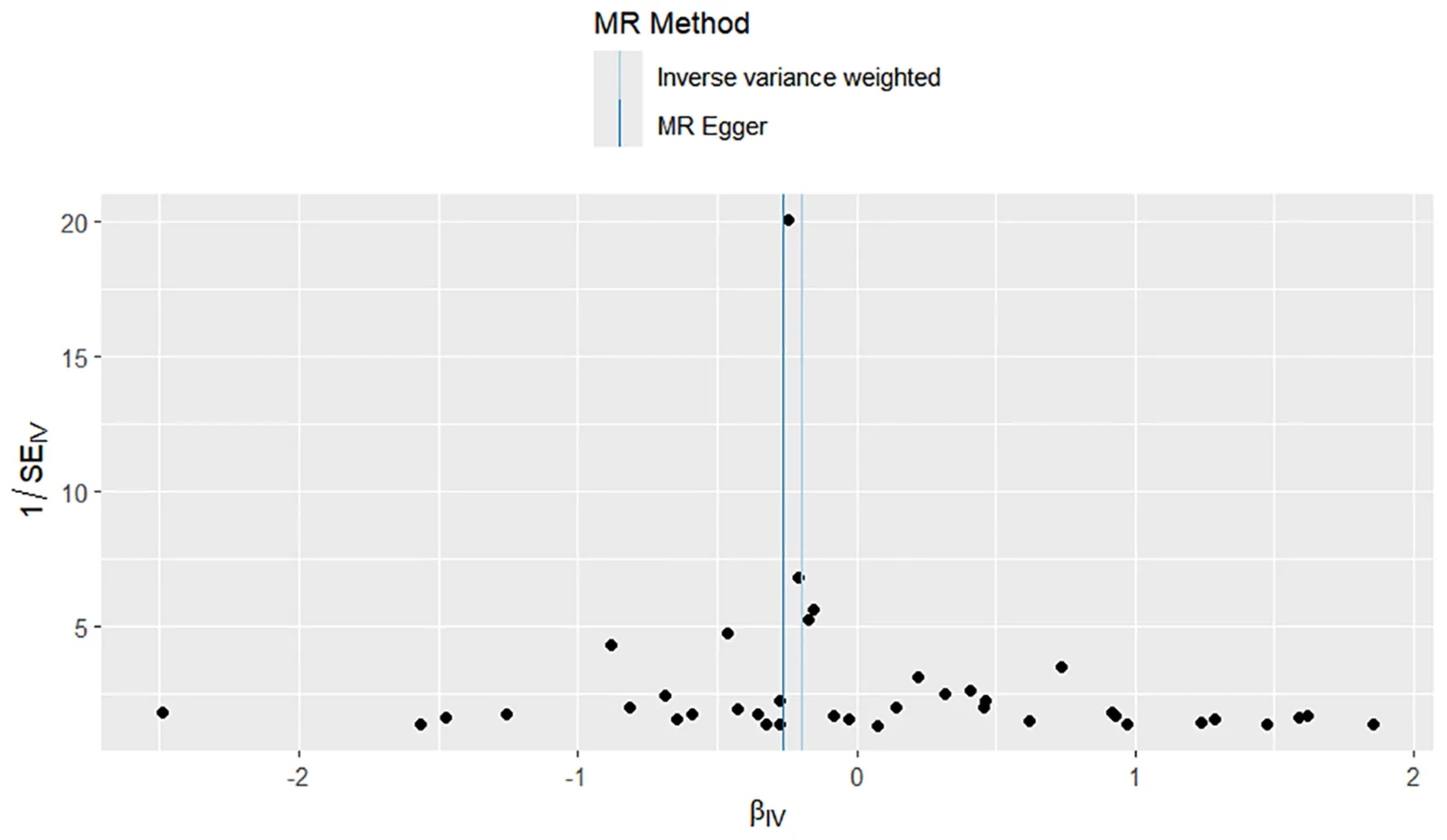
Funnel plot of the association between genetically predicted omega-3 fatty acids and Crohn’s disease.

**Figure 7.**
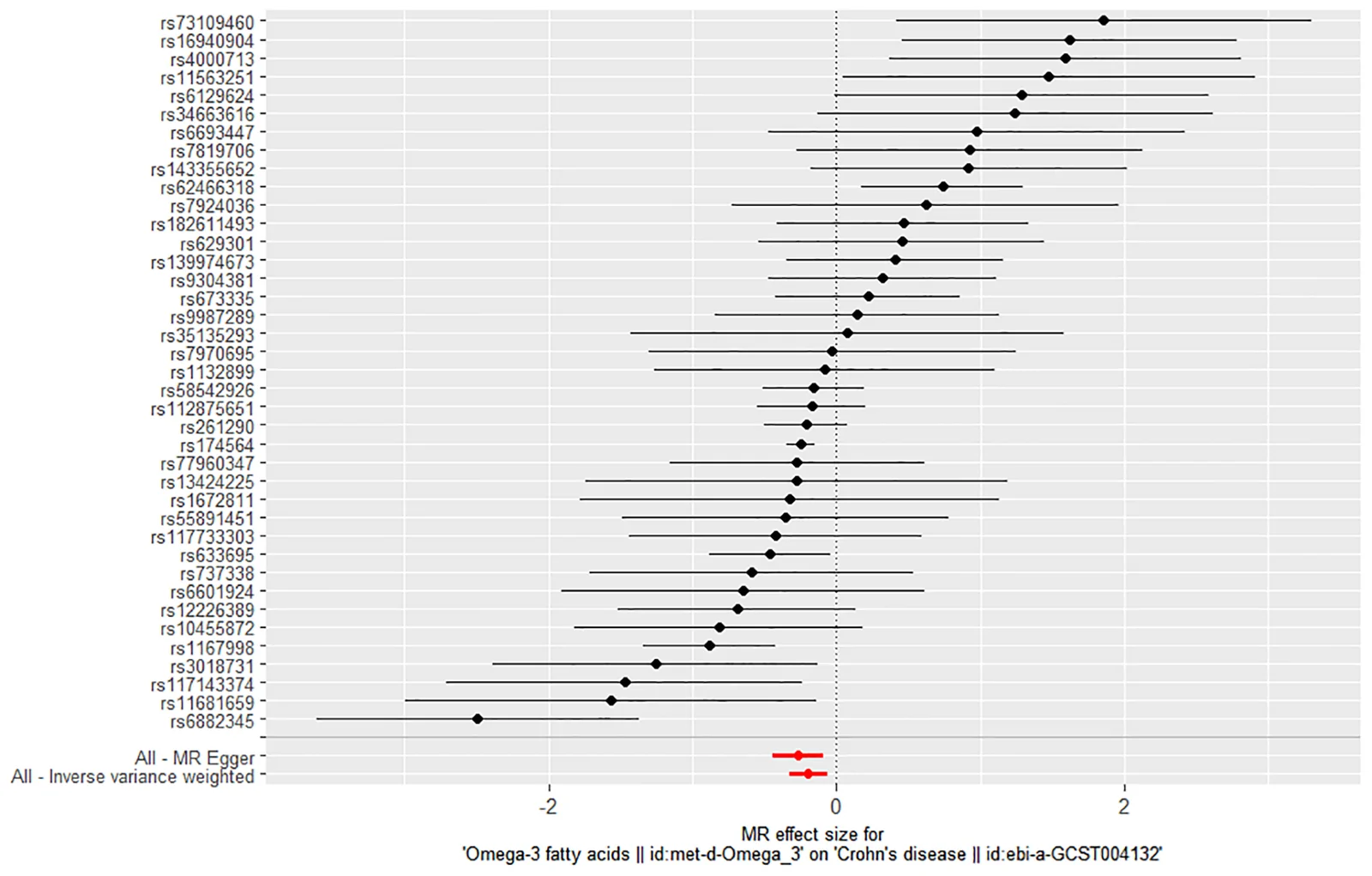
Forest plot of the association between genetically predicted omega-3 fatty acids and Crohn’s disease.

### 3.2. Relationship between omega-6 fatty acids and Crohn’s disease

#### 3.2.1. Instrumental-variable results

Sixty-three exposure-associated, independent SNPs were initially included. Four were lost during harmonization, and eight palindromic or incompatible SNPs (rs1883711, rs3817335, rs4704210, rs4766578, rs4860948, rs740516, rs9616847, rs964184) were excluded, leaving 51 SNPs for analysis (Table 2). All F values exceeded 10; the range was 26.909 to 545.974, with a mean of 115.406, indicating no weak-instrument bias.

**Table 2:** A total of 51 independent SNPs strongly associated with the exposure were included for Omega-6 fatty acids.

SNP	effect	other	beta	se	chr	eaf	pval	F_value
rs1002687	A	G	0.090971	0.00421287	1	0.644748	1E–107	466.2832572
rs1081105	C	A	0.118778	0.0123322	19	0.027624	1.8E–22	92.76644158
rs112875651	A	G	−0.063694	0.00418831	8	0.392346	2.2E–53	231.2700582
rs114863007	A	G	−0.0457294	0.00693029	6	0.094543	7.3E–12	43.53997647
rs12740374	T	G	−0.0572469	0.00485222	1	0.221071	1.5E–32	139.1947902
rs13108218	G	A	−0.035165	0.00417574	4	0.615417	3.6E–18	70.91765132
rs141469619	G	A	0.110551	0.0213113	11	0.01014	0.000000014	26.9094899
rs142158911	A	G	−0.0944019	0.00631781	19	0.116705	5.2E–52	223.2689597
rs1461729	G	A	0.0835465	0.00670616	8	0.899221	2.8E–36	155.2060691
rs1800961	T	C	−0.0744567	0.0117812	20	0.030192	3.3E–10	39.94188072
rs183130	T	C	0.0615133	0.00431123	16	0.324072	1.4E–48	203.5802611
rs1883711	C	G	0.0922513	0.0118528	20	0.031191	3.2E–16	60.5763466
rs2378390	A	G	−0.0333321	0.00581966	20	0.140871	3.2E–09	32.80425103
rs261290	C	T	−0.0965507	0.00425095	15	0.654653	1E–116	515.8683221
rs2737245	T	G	−0.0274764	0.00450795	8	0.278598	1.4E–09	37.15022766
rs2740488	C	A	−0.0501345	0.00457926	9	0.265321	5.4E–28	119.8623453
rs28383314	C	T	0.0388549	0.00415994	6	0.622939	1.7E–18	87.24036174
rs3734854	A	G	0.0477116	0.0082133	6	0.064679	5.9E–11	33.74524528
rs3770586	T	C	−0.0229674	0.00405156	2	0.48397	7.1E–09	32.13506026
rs3817335	A	T	−0.0277621	0.00421464	11	0.350559	9.8E–12	43.38940146
rs4008004	A	C	0.0329594	0.00487542	9	0.221842	8.4E–12	45.70192835
rs4299376	T	G	−0.0353905	0.00431976	2	0.676279	1.1E–16	67.12034182
rs4439799	T	C	0.0224228	0.00404559	17	0.502274	0.000000013	30.719628
rs4704210	C	G	0.0467287	0.00416788	5	0.374212	6.1E–30	125.7004942
rs4766578	A	T	0.0277794	0.00404125	12	0.503267	1.5E–12	47.25135721
rs4860948	A	T	0.027907	0.00470731	4	0.244353	1.9E–09	35.1463885
rs534417	G	A	0.0390397	0.00607823	1	0.875005	9.3E–11	41.25329894
rs55747707	A	G	−0.0494876	0.0050239	7	0.203615	1.7E–22	97.03106813
rs56322906	A	G	−0.100371	0.0109592	19	0.035157	1.2E–19	83.88007243
rs58542926	T	C	−0.128057	0.0076883	19	0.074383	2.5E–65	277.4252615
rs633695	G	A	0.0725178	0.00445048	15	0.292348	1.3E–59	265.5067295
rs6471717	A	G	−0.0290285	0.00427754	8	0.663091	4E–12	46.05332923
rs6547409	T	C	−0.0813514	0.00924809	2	0.051258	2.4E–20	77.3795086
rs6602911	T	C	0.0257493	0.00420044	13	0.360079	1.3E–09	37.57865944
rs672889	G	T	0.0763943	0.00580512	2	0.860091	1.3E–41	173.180707
rs6882345	A	G	0.0448784	0.00417905	5	0.632863	1.2E–27	115.323973
rs6934962	T	C	0.0228258	0.00411241	6	0.400489	0.000000022	30.80769473
rs6938647	C	A	−0.0481657	0.00494853	6	0.782336	1.9E–23	94.73780925
rs7139079	A	G	−0.0297379	0.00411541	12	0.592501	3.3E–13	52.21489108
rs72997616	A	C	−0.051564	0.00694288	11	0.094064	1.6E–13	55.15868214
rs740516	G	C	−0.0315718	0.00565892	17	0.151132	0.000000014	31.1265923
rs75406471	A	G	−0.0314077	0.00559075	10	0.154684	0.000000027	31.55964599
rs7707394	A	G	0.02951	0.00420247	5	0.357106	1.2E–12	49.30933838
rs7750288	G	A	0.0249212	0.00446247	6	0.285011	0.000000013	31.18798252
rs77960347	G	A	0.275526	0.0176159	18	0.013239	2.8E–56	244.6332596
rs79429216	A	G	0.15083	0.0179625	19	0.012713	1.3E–17	70.50856887
rs870526	T	C	−0.0318914	0.00403955	2	0.521426	7.7E–16	62.32771327
rs9295128	T	G	−0.196375	0.0159543	6	0.016833	3.4E–36	151.5014843
rs9304381	T	C	0.0702336	0.00523609	18	0.818434	7.2E–42	179.9184306
rs9616847	T	A	0.023821	0.00415851	22	0.388327	0.000000014	32.81288964
rs964184	C	G	−0.138896	0.00594434	11	0.867229	1.1E–125	545.9742922

#### 3.2.2. MR analysis results

The multiplicative random-effects IVW analysis showed no statistically significant association between genetically predicted omega-6 fatty acids and CD [OR = 0.885, 95% CI 0.727–1.061, *P* = .186]. Accordingly, the available genetic data did not provide evidence for an association of circulating omega-6 alone with CD susceptibility (Figs. 8 and 9).

**Figure 8.**
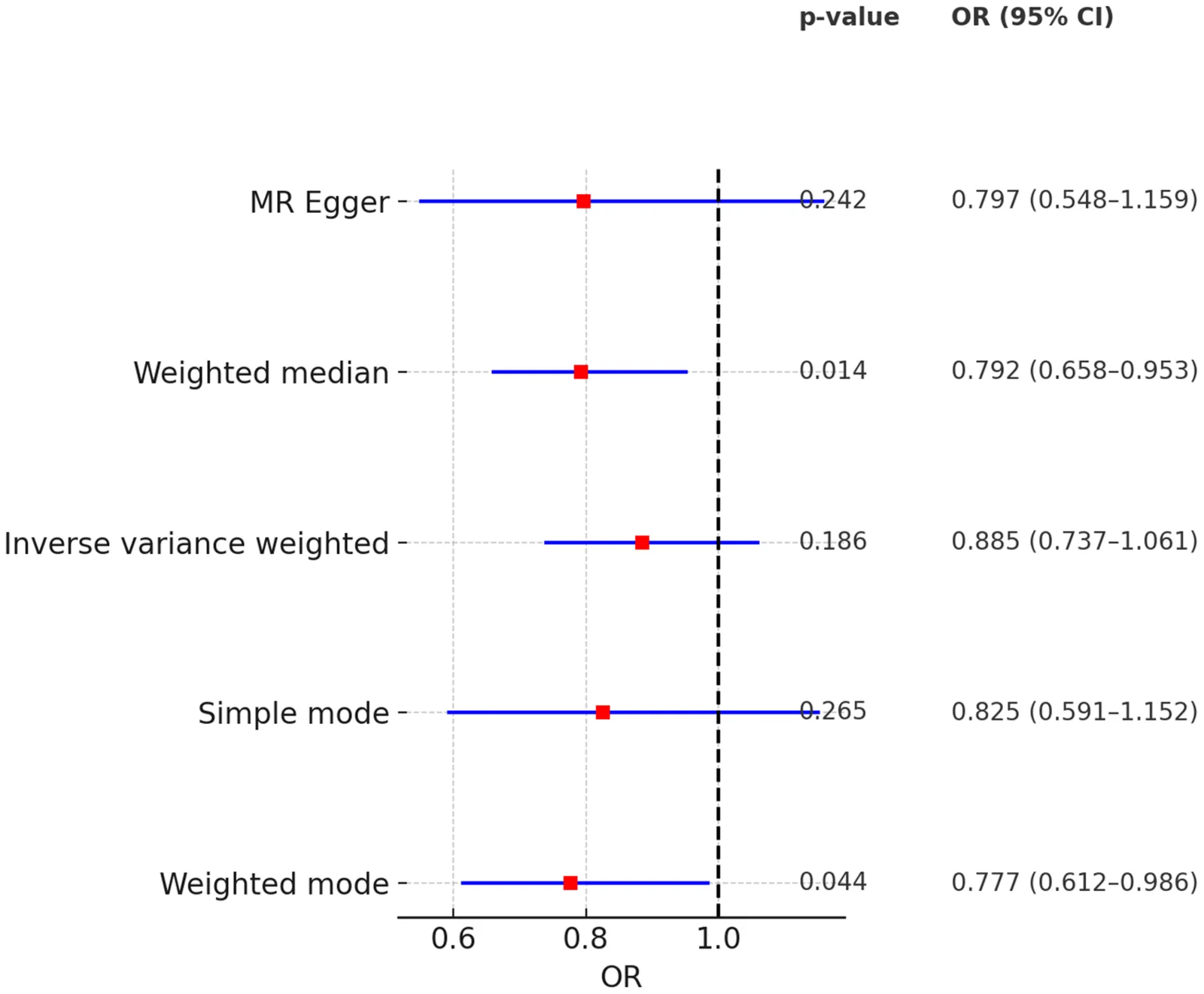
MR estimates for the association between genetically predicted omega-6 fatty acids and Crohn’s disease.

**Figure 9.**
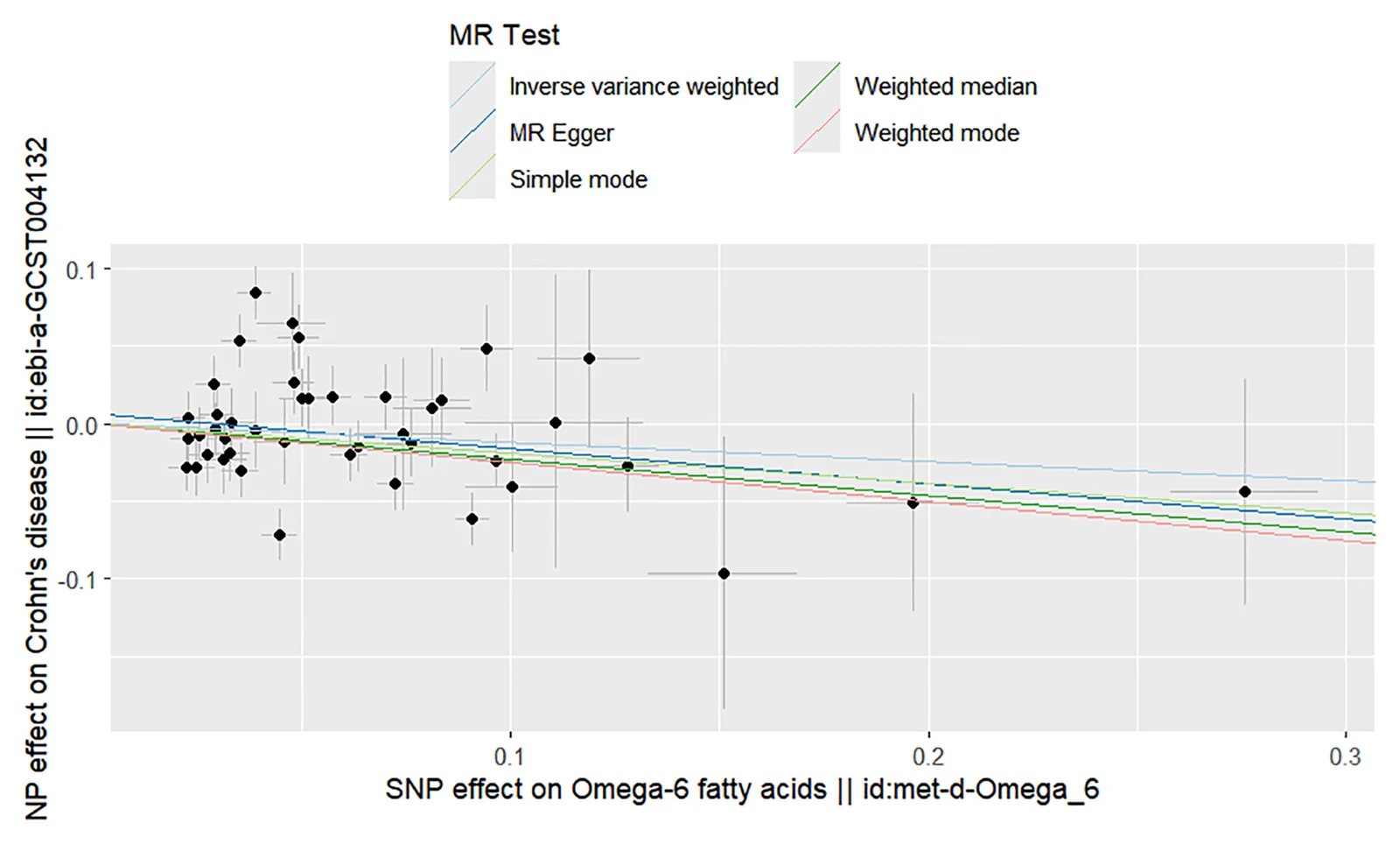
Scatter plot of the association between genetically predicted omega-6 fatty acids and Crohn’s disease.

#### 3.2.3. Sensitivity analysis

Cochran’s Q test indicated substantial between-instrument heterogeneity (IVW: Q = 109.273, *P* < .001; MR-Egger: Q = 108.239, *P* < .001). Although the multiplicative random-effects IVW model accounts for over-dispersion when estimating the uncertainty of the pooled association, it does not eliminate potential bias arising from heterogeneous biological pathways or invalid instruments. The MR-Egger intercept was 0.007 (*P* = .535), providing no statistical evidence of directional horizontal pleiotropy. Leave-one-out analysis did not identify a single influential SNP (Fig. 10), and the funnel plot was broadly symmetrical (Fig. 11). Accordingly, the absence of a statistically significant pooled association should not be interpreted as evidence that omega-6 has no biological relevance to CD, particularly in the presence of substantial between-instrument heterogeneity. The forest plot is shown in Figure 12.

**Figure 10.**
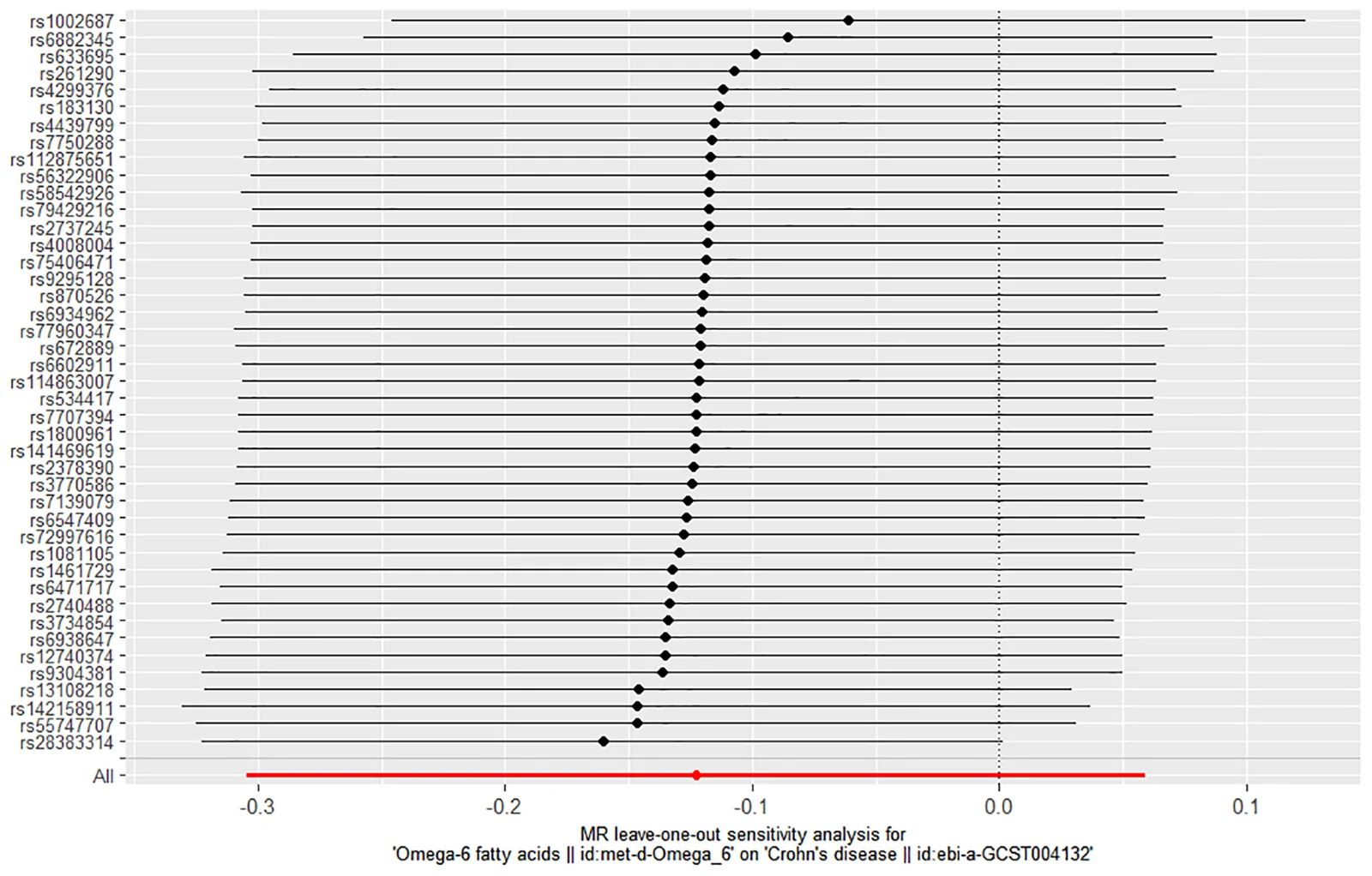
Leave-one-out analysis of the association between genetically predicted omega-6 fatty acids and Crohn’s disease.

**Figure 11.**
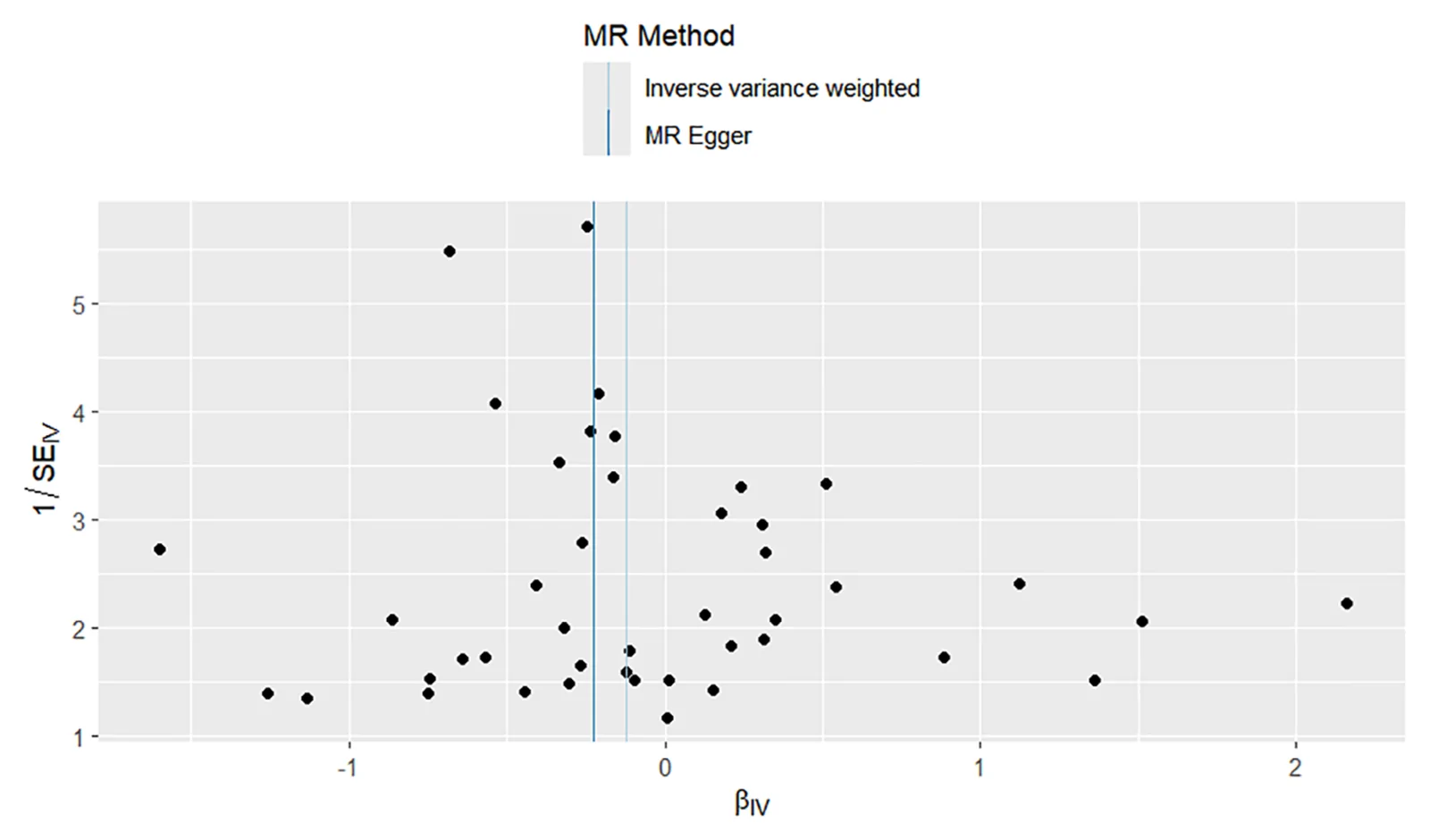
Funnel plot of the association between genetically predicted omega-6 fatty acids and Crohn’s disease.

**Figure 12.**
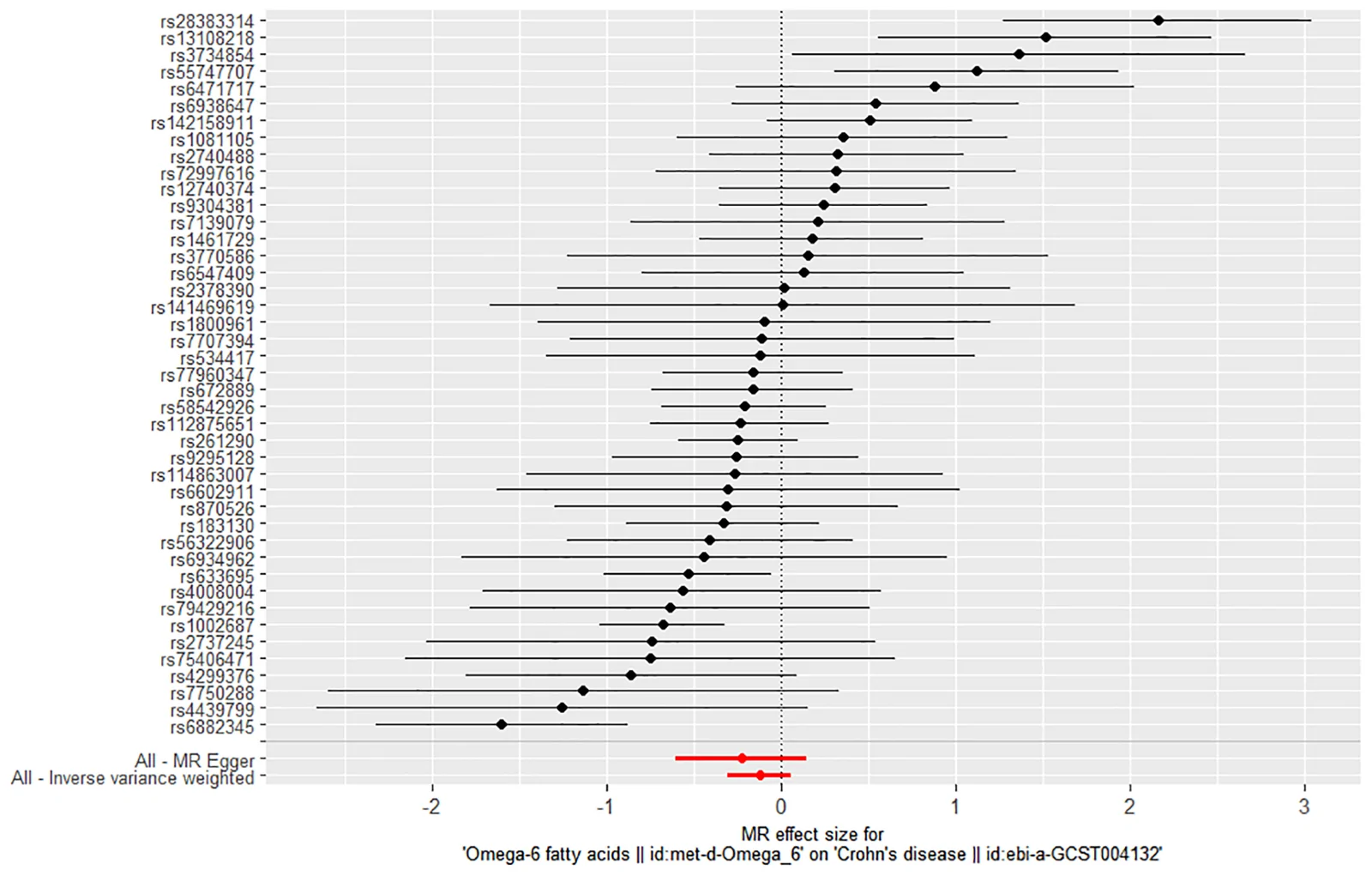
Forest plot of the association between genetically predicted omega-6 fatty acids and Crohn’s disease.

### 3.3. Relationship between the omega-6/omega-3 fatty-acid ratio and Crohn’s disease

#### 3.3.1. Instrumental-variable results

Forty-one exposure-associated, independent SNPs were initially included. Two were lost during harmonization, and five palindromic or incompatible SNPs (rs12976395, rs4860987, rs72789541, rs7916868, rs964184) were excluded, leaving 34 SNPs for analysis (Table 3). All F values were above 10; the range was 28.311 to 7582.712, with a mean of 320.687, indicating no weak-instrument bias.

**Table 3:** A total of 34 independent SNPs strongly associated with the exposure were included for Omega-6/Omega-3 fatty acid ratio.

SNP	effect	other	beta	se	chr	eaf	pval	F_value
rs10733306	T	C	0.0225443	0.00413317	9	0.46003	0.000000032	29.75137028
rs112875651	A	G	0.0718479	0.00424549	8	0.392346	6.5E–65	286.3996701
rs11632618	A	G	−0.0815347	0.00800529	15	0.069927	1.2E–24	103.736315
rs117143374	C	T	0.0343687	0.00587978	21	0.142254	0.000000006	34.16678046
rs12226389	C	T	0.0563519	0.00527721	11	0.18582	8.4E–27	114.0271773
rs12976395	C	G	−0.0274747	0.00439626	19	0.504524	7.5E–10	39.05702679
rs13273454	T	C	0.0233472	0.00411509	8	0.470545	5.8E–09	32.18925544
rs139974673	C	T	−0.117385	0.0128859	15	0.025918	9.1E–21	82.98425418
rs143355652	T	C	0.16027	0.0205193	11	0.010467	7.6E–15	61.00696102
rs145659493	A	C	−0.115733	0.0163525	11	0.015854	4.3E–13	50.08943192
rs145786300	A	G	0.174042	0.0193409	11	0.011985	1.1E–19	80.97571133
rs149820547	G	T	0.0653372	0.0102102	11	0.042103	1.2E–10	40.94987115
rs1560390	C	T	0.0352816	0.00496671	15	0.219684	2.8E–13	50.46135849
rs1564348	C	T	0.030467	0.00544576	6	0.169745	0.000000014	31.29985963
rs16940904	T	C	0.0412048	0.00490538	17	0.226571	2.3E–18	70.55865748
rs174564	G	A	0.371404	0.00426515	11	0.347013	1E–200	7582.711537
rs182611493	G	A	0.18661	0.0196926	19	0.012519	1.1E–21	89.79738852
rs2232143	C	T	−0.10962	0.0145796	11	0.021879	9.4E–14	56.53122252
rs261291	C	T	−0.0891379	0.00427499	15	0.356068	9.9E–99	434.7650156
rs4000713	A	G	0.0308836	0.00448656	7	0.295408	6.1E–13	47.38368993
rs4860987	T	A	−0.0426258	0.00493905	4	0.258609	6.8E–18	74.48318517
rs55891451	C	A	−0.0355372	0.0051102	10	0.201728	1.2E–12	48.36048166
rs58542926	T	C	0.143289	0.00779781	19	0.074383	3.4E–79	337.6606137
rs62466318	T	C	0.0606132	0.00509341	7	0.204178	4.4E–33	141.6175746
rs638714	T	G	0.0438817	0.00431435	1	0.345963	3.7E–25	103.4513446
rs6698680	G	A	−0.0258639	0.00410413	1	0.461958	2.1E–11	39.71419897
rs673335	C	T	0.0598626	0.00557189	11	0.159762	7.4E–28	115.4266478
rs7222755	G	A	0.0239677	0.00450454	17	0.290719	0.000000041	28.31077932
rs72789541	A	T	0.0881716	0.00448603	16	0.29597	1.4E–86	386.3074613
rs73109460	A	G	0.0338895	0.00625247	7	0.123622	2.2E–09	29.37832896
rs7916868	T	A	−0.0295041	0.00409087	10	0.504491	5.1E–14	52.0155728
rs8074191	C	T	0.0277573	0.00476891	17	0.756127	4.8E–09	33.87787691
rs964184	C	G	0.0735236	0.00599779	11	0.867229	5.3E–35	150.2695603
rs9947684	G	A	−0.0283663	0.00429465	18	0.654493	5.7E–12	43.62644563

#### 3.3.2. MR analysis results

The multiplicative random-effects IVW analysis showed that a higher genetically predicted omega-6/omega-3 ratio was associated with higher odds of CD [OR = 1.211, 95% CI 1.069–1.372, *P* = .003]. MR-Egger, weighted median, and simple mode estimates were directionally consistent and statistically significant (*P* < .05), supporting an association between genetically predicted PUFA balance and CD susceptibility (Figs. 13 and 14).

**Figure 13.**
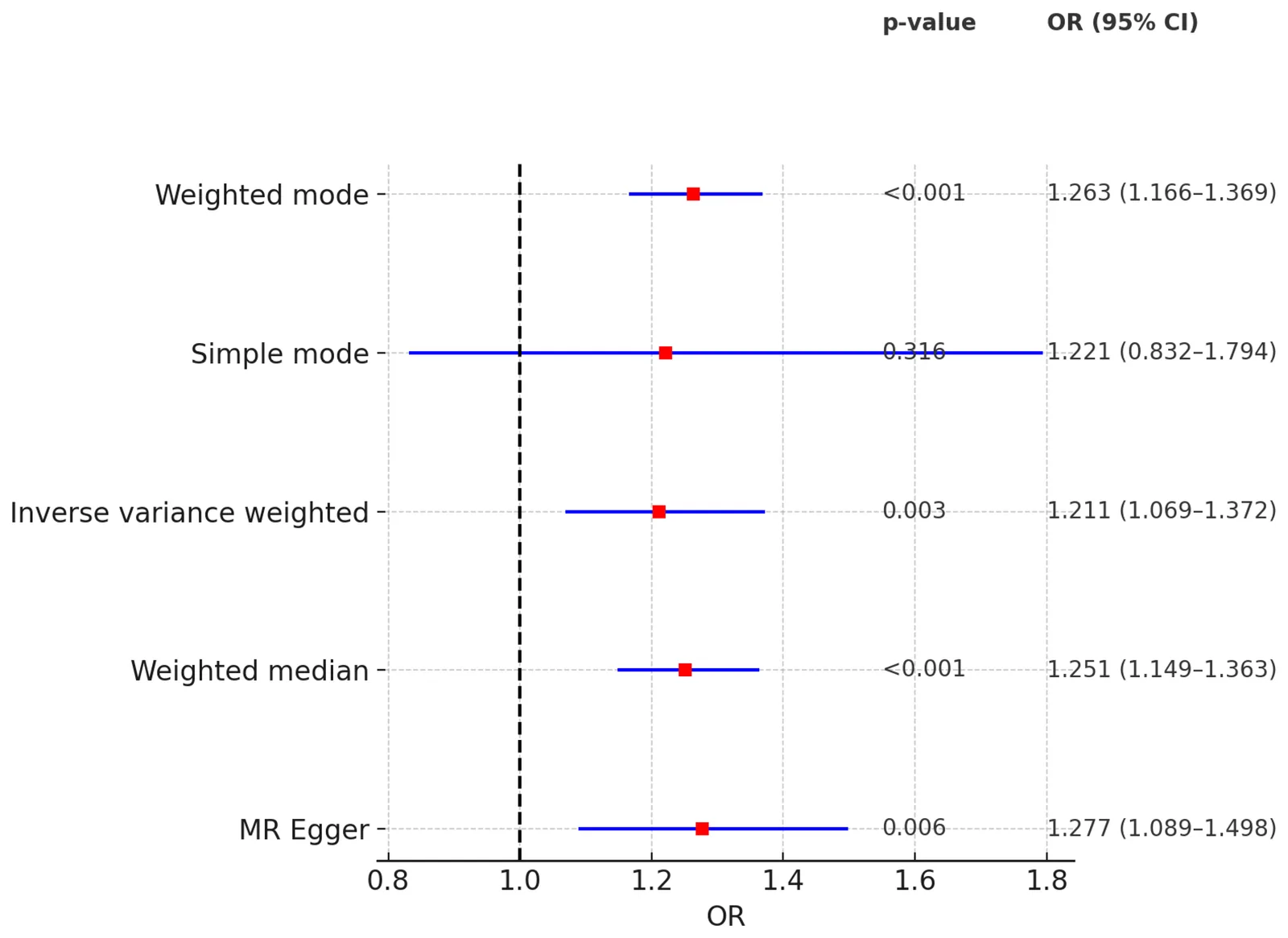
MR estimates for the association between the genetically predicted omega-6/omega-3 fatty acid ratio and Crohn’s disease.

**Figure 14.**
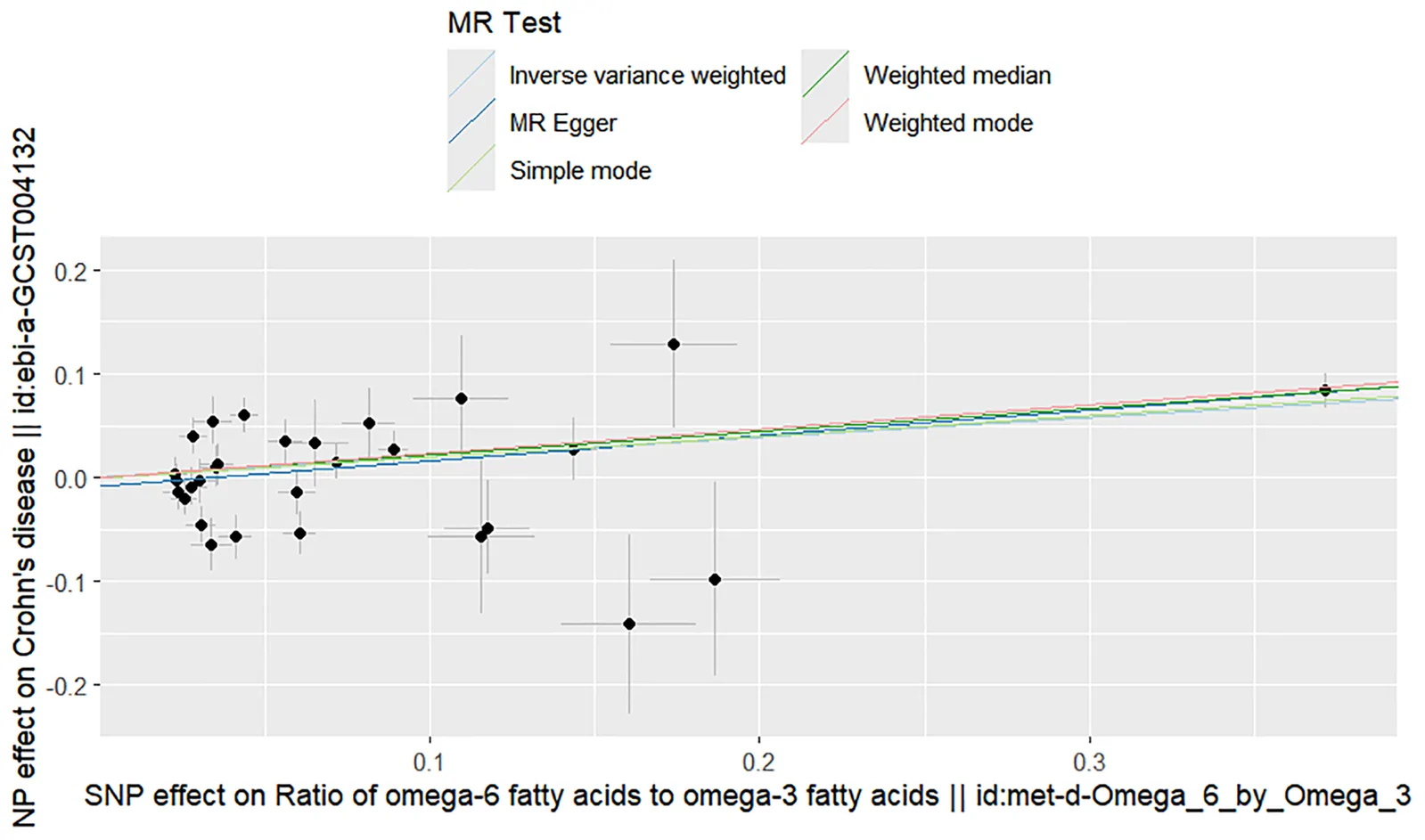
Scatter plot of the association between the genetically predicted omega-6/omega-3 fatty acid ratio and Crohn’s disease.

#### 3.3.3. Sensitivity analysis

Cochran’s Q test showed substantial heterogeneity for the omega-6/omega-3 ratio (IVW: Q = 74.402, *P* < .001; MR-Egger: Q = 71.489, *P* < .001). Thus, the pooled estimate should not be interpreted as a homogeneous effect across all genetic instruments. Although the multiplicative random-effects IVW model accounts for over-dispersion in estimating the standard error, residual biological heterogeneity and horizontal pleiotropy may remain. The MR-Egger intercept was − 0.009 (*P* = .304), providing no statistical evidence of directional horizontal pleiotropy. Leave-one-out analysis did not identify a single SNP driving the association (Fig. 15), and the funnel plot was broadly symmetrical (Fig. 16). These findings support robustness against single-variant influence but do not eliminate the uncertainty introduced by substantial between-instrument heterogeneity. The forest plot is shown in Figure 17.

**Figure 15.**
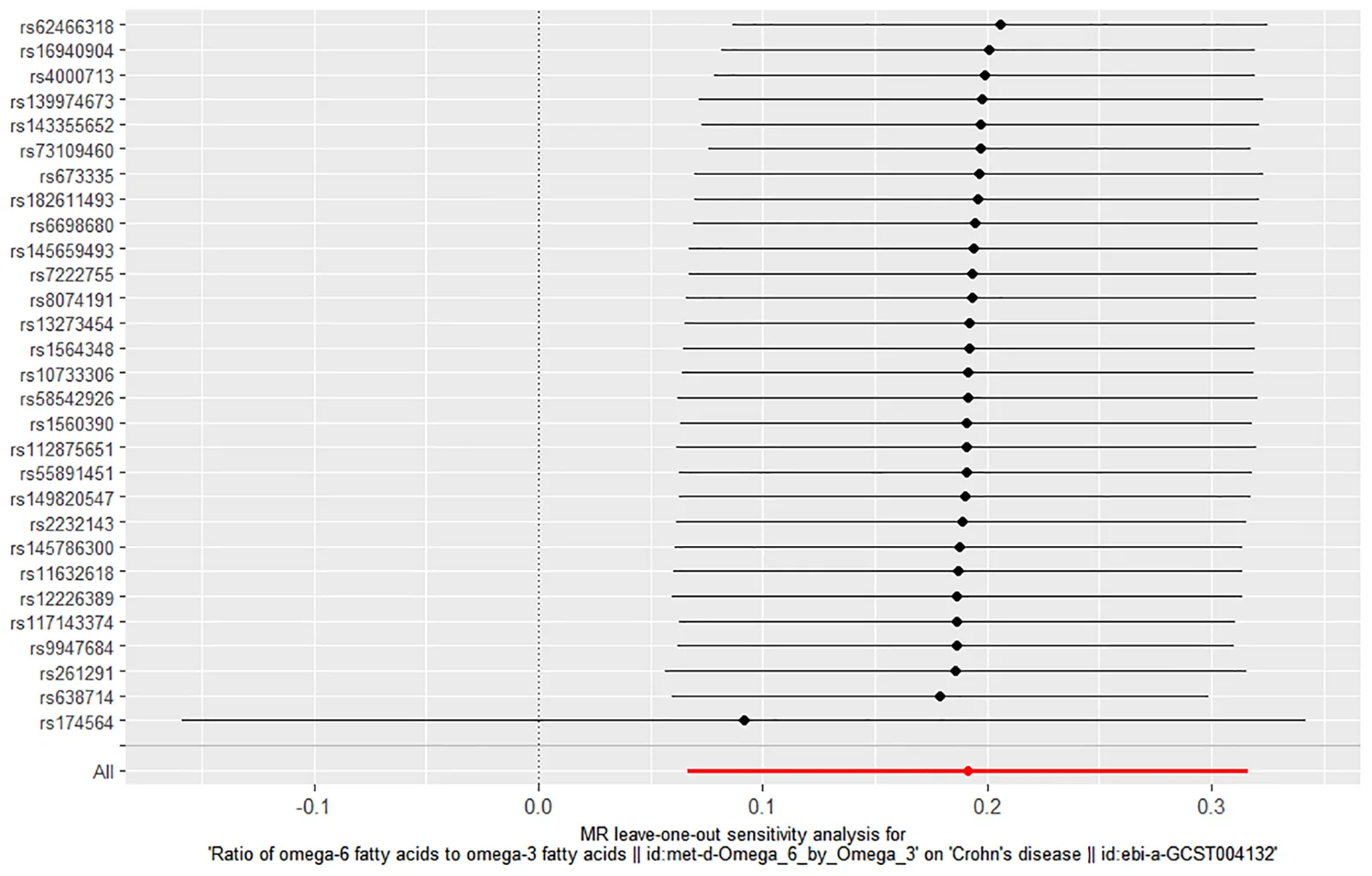
Leave-one-out analysis of the association between the genetically predicted omega-6/omega-3 fatty acid ratio and Crohn’s disease.

**Figure 16.**
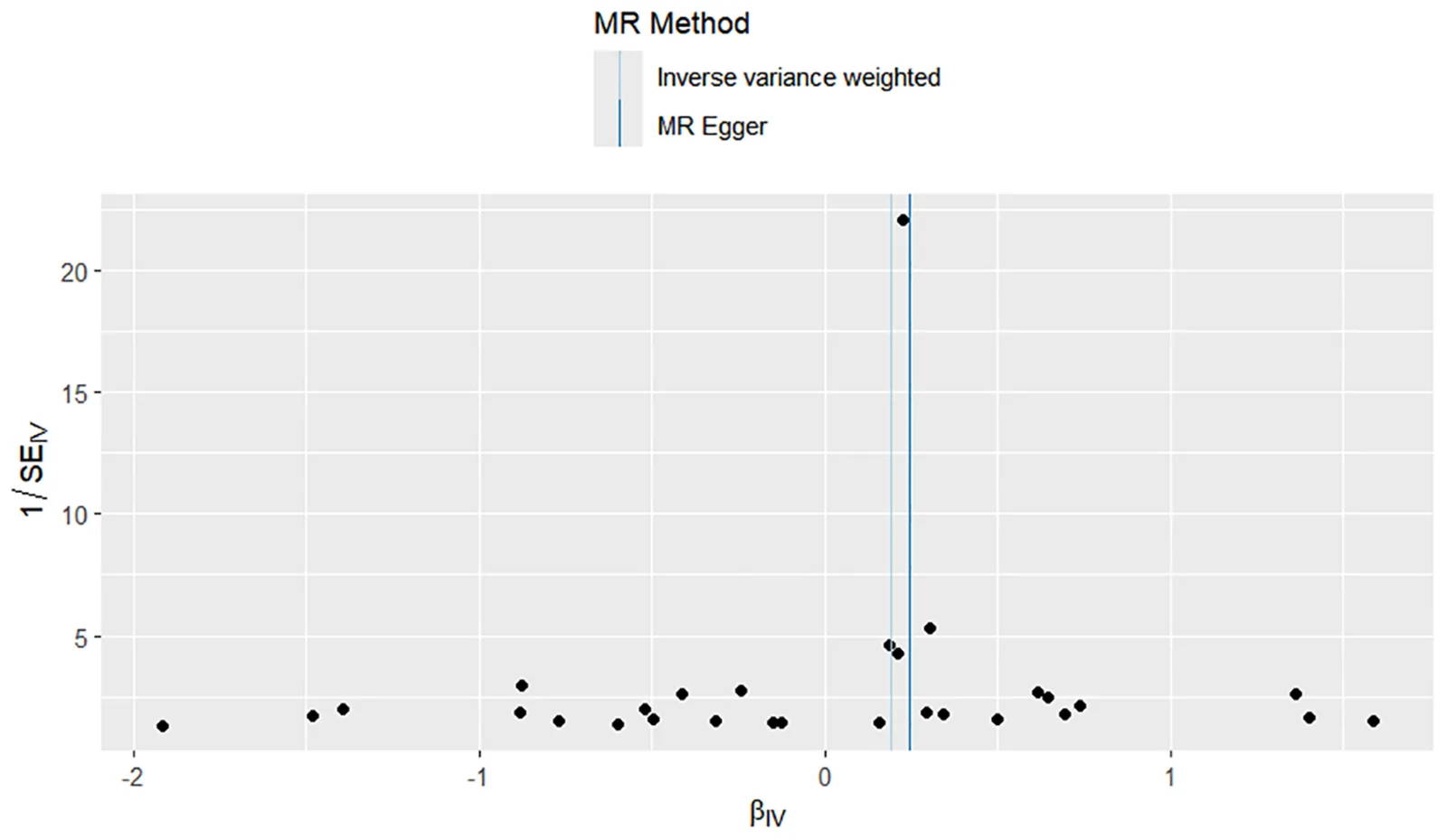
Funnel plot of the association between the genetically predicted omega-6/omega-3 fatty acid ratio and Crohn’s disease.

**Figure 17.**
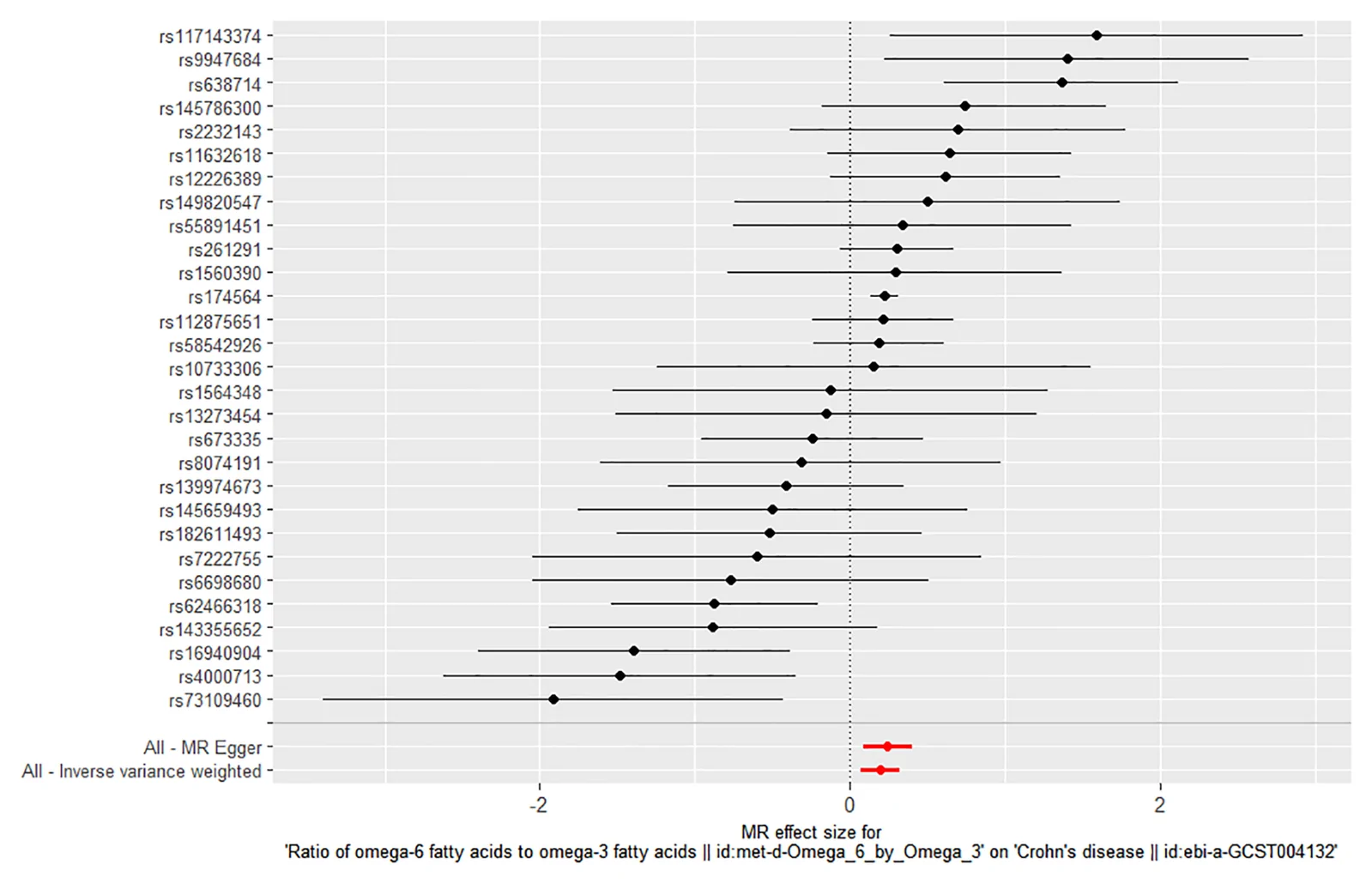
Forest plot of the association between the genetically predicted omega-6/omega-3 fatty acid ratio and Crohn’s disease.

## 4. Discussion

In this two-sample MR study, genetically predicted higher circulating omega-3 was associated with lower odds of CD, omega-6 alone showed no statistically significant association, and a higher omega-6/omega-3 ratio was associated with higher CD odds. These findings provide genetic evidence linking circulating PUFA profiles with CD susceptibility. However, substantial between-instrument heterogeneity limits the strength of causal inference, and the results should not be interpreted as definitive evidence that altering dietary omega-3 intake or taking fish-oil supplements prevents CD. The exposure captured by MR represents lifelong genetically influenced differences in circulating PUFA traits, which are biologically and temporally distinct from short-term dietary supplementation.

A central issue raised by the present analysis is whether the selected SNPs are genuinely related to circulating omega-3 levels. The genetic relevance of the selected SNPs to circulating omega-3 levels is supported by large-scale GWAS evidence. The exposure dataset was derived from a UK Biobank metabolomic GWAS of more than 114,000 participants, in which dozens of independent genome-wide significant variants were associated with circulating PUFA traits and explained measurable phenotypic variance.^[11]^ Earlier GWAS also identified biologically plausible loci in fatty-acid metabolism, particularly FADS1/FADS2 and ELOVL2, that are strongly associated with circulating long-chain omega-3 species.^[14]^ In our omega-3 instrument set, rs174564, located in the FADS region, showed a particularly strong exposure association. FADS enzymes catalyze key desaturation steps in the conversion of essential fatty-acid precursors, providing a plausible biological route from genotype to circulating PUFA composition.^[14,15]^

Nevertheless, a genome-wide association between a SNP and omega-3 concentration does not prove that the variant acts exclusively or directly through omega-3. Genetic variants can influence circulating PUFA concentrations through desaturation, elongation, hepatic lipid handling, lipoprotein transport, or other metabolic pathways, and some of these pathways may independently affect inflammatory disease. This distinction is important for the MR exclusion-restriction assumption. Indeed, analyses of the same large UK Biobank PUFA GWAS in other disease settings have shown that estimates can change after accounting for lipoprotein-related traits or excluding variants near the FADS locus, illustrating the potential for correlated lipid pathways and horizontal pleiotropy.^[11]^ Therefore, our results are most appropriately interpreted as evidence based on genetically proxied circulating PUFA traits, not as proof of a single molecular pathway. Accordingly, the present analysis supports the relevance of the selected variants as statistical instruments for circulating PUFA traits but cannot establish that every instrument influences CD exclusively through omega-3 or omega-6 metabolism. This limitation is particularly important when interpreting the findings causally.

Dietary intake and supplementation add a second layer of complexity. Circulating omega-3 is influenced by both environmental exposure and endogenous metabolism; genetic variation affects absorption, conversion, tissue handling, and baseline concentrations, while diet can modify the same biomarker. The random allocation of alleles at conception helps reduce confounding by adult dietary choices, but MR does not assume that diet is irrelevant. Rather, it estimates the association with the genetically determined component of circulating omega-3. Gene–diet interaction is also biologically plausible: FADS variants can modify the relationship between PUFA intake and circulating fatty-acid profiles, and previous work in CD has reported interactions between dietary PUFA ratios and FADS variants.^[16]^ Thus, an MR estimate cannot be translated directly into a recommended dose of fish, omega-3 supplements, or an optimal dietary omega-6/omega-3 ratio. Therefore, the present MR findings have no direct implication for the dose, formulation, duration, or clinical effectiveness of omega-3 supplementation. At present, they should not be used to guide dietary prevention or therapeutic recommendations for CD.

Our findings should also be viewed alongside the mixed clinical literature. Earlier studies suggested possible benefits of omega-3 preparations, whereas large randomized trials and systematic reviews did not demonstrate a consistent benefit of fish-oil supplementation for maintaining remission in CD.^[17–20]^ Previous MR studies have reported inverse associations between genetically predicted omega-3-related traits and IBD or CD, including signals involving EPA and FADS-region variants.^[15,21]^ These apparently different lines of evidence are not necessarily contradictory because genetic proxies represent lifelong metabolic differences, whereas randomized supplementation trials test specific formulations, doses, treatment durations, disease stages, and adherence patterns. The current MR study therefore contributes suggestive etiologic evidence regarding genetically proxied PUFA traits but does not establish clinical efficacy.

The substantial Cochran’s Q statistics represent an important limitation of the present analysis. For omega-3, significant heterogeneity was observed under both the IVW model (Q = 114.548, *P* < .001) and MR-Egger regression (Q = 110.620, *P* < .001). We emphasize that this heterogeneity does affect the certainty of our final interpretation. It indicates considerable variability among SNP-specific estimates and raises the possibility that the genetic instruments operate through heterogeneous biological, metabolic, or pleiotropic pathways. We used a multiplicative random-effects IVW estimator because this approach allows for over-dispersion among SNP-specific estimates and adjusts the uncertainty of the pooled estimate when heterogeneity is present. Nevertheless, random-effects modeling cannot correct bias from invalid genetic instruments and does not eliminate horizontal pleiotropy. The nonsignificant MR-Egger intercepts, broadly symmetrical funnel plots, directionally concordant complementary estimators, and leave-one-out analyses provide some reassurance against strong directional pleiotropy or a single dominant variant. However, none of these analyses can fully exclude balanced pleiotropy, correlated lipid pathways, or other sources of biological heterogeneity. Consequently, we do not interpret the significant IVW estimate as definitive evidence of a homogeneous causal effect. Rather, the omega-3 association should be regarded as suggestive genetic evidence that is compatible with, but does not prove, a causal contribution to CD susceptibility.

Mechanistically, long-chain omega-3 PUFAs can give rise to specialized pro-resolving mediators such as resolvins and protectins that attenuate inflammatory signaling and support epithelial-barrier recovery.^[22,23]^ Conversely, arachidonic-acid-derived mediators may promote inflammatory responses under some conditions, although the biology of omega-6 fatty acids is heterogeneous and cannot be reduced to a uniformly pro-inflammatory effect. The FADS pathway is particularly relevant because it influences the conversion of precursor fatty acids into downstream long-chain products and has been implicated in CD-related inflammatory biology.^[16,24]^ These mechanisms provide biological plausibility, but the present MR analysis does not identify which specific omega-3 species, metabolic step, or inflammatory mediator is responsible for the observed association.

Several limitations should be acknowledged. First, all GWAS participants were of European ancestry, which limits the generalizability of the findings to other populations. Second, the exposure traits represent aggregate circulating omega-3 and omega-6 measures rather than individual PUFA species; therefore, the effects of EPA, DHA, alpha-linolenic acid, linoleic acid, and arachidonic acid cannot be distinguished. Third, substantial between-instrument heterogeneity was observed for all three exposures, raising the possibility of residual horizontal pleiotropy, correlated lipid pathways, or pathway-specific genetic effects despite nonsignificant MR-Egger intercept tests. Fourth, summary-level data prevented assessment of nonlinear associations, detailed gene–diet interactions, disease phenotype, age at onset, or treatment status. Finally, MR estimates represent lifelong genetically proxied differences in circulating PUFA traits and should not be interpreted as estimates of the clinical benefit of dietary supplementation. Future studies incorporating individual PUFA species, pleiotropy-robust approaches, multivariable MR, colocalization or locus-specific analyses, diverse ancestries, detailed dietary information, and prospective intervention studies are needed.

## 5. Conclusion

Using large-scale GWAS summary data, this two-sample MR study identified an inverse association between genetically predicted higher circulating omega-3 and CD susceptibility, no statistically significant association for omega-6 alone, and a positive association between a genetically predicted higher omega-6/omega-3 ratio and CD susceptibility. However, substantial between-instrument heterogeneity and the possibility of residual horizontal pleiotropy limit the strength of causal inference. Accordingly, these findings should be regarded as suggestive genetic evidence linking circulating PUFA profiles with CD susceptibility rather than definitive proof of causality. They do not establish that dietary omega-3 intake or supplementation prevents CD and should not be translated directly into clinical or nutritional recommendations. Further validation using individual PUFA species, pleiotropy-robust and multivariable genetic analyses, diverse populations, and prospective intervention studies is required.

## Author contributions

**Conceptualization:** Tingting Zhu.

**Data curation:** Tingting Zhu, Kaifeng Zhu.

**Writing – review & editing:** Tingting Zhu, Qiang Hu, Kaifeng Zhu.

**Formal analysis:** Kaifeng Zhu.

**Writing – original draft:** Kaifeng Zhu.
